# NNT-AS1 in CAFs-derived exosomes promotes progression and glucose metabolism through miR-889-3p/HIF-1α in pancreatic adenocarcinoma

**DOI:** 10.1038/s41598-024-57769-6

**Published:** 2024-03-24

**Authors:** Pingping Zhang, Qun Wang, Weijun Lu, Feng Zhang, Dongde Wu, Junwei Sun

**Affiliations:** 1 Department of Radiation Oncology, Hubei Cancer Hospital, Tongji Medical College, Huazhong University of Science and Technology, Wuhan, China; 2 Department of Hepatic & Biliary & Pancreatic Surgery, Hubei Cancer Hospital, Tongji Medical College, Huazhong University of Science and Technology, Wuhan, China

**Keywords:** CAFs-derived exosomes, NNT-AS1, PDAC, miR-889-3p, HIF-1α, Glucose metabolism, Molecular biology, Medical research, Oncology

## Abstract

It is metabolic and signaling crosstalk between stromal cells and tumors in the tumor microenvironment, which influences several aspects of tumor formation and drug resistance, including metabolic reprogramming. Despite considerable findings linking lncRNAs in HIF-1-related regulatory networks to cancer cell, little emphasis has been given to the role in communication between cancer-associated fibroblasts (CAFs) and tumor cells. Previously, we observed that NNT-AS1 was substantially expressed in CAFs cells and CAFs exosomes, and subsequently investigated the influence of CAFs exosomal NNT-AS1 on glucose metabolism, proliferation, and metastasis of pancreatic ductal adenocarcinoma (PDAC) cells. Transmission electron microscopy was used to examine exosomes secreted by PDAC patient-derived CAFs. qRT-PCR was used to evaluate the expression of NNT-AS1, miR-889-3p, and HIF-1. The role of CAFs-derived exosomal NNT-AS1 in PDAC cell progression and metabolism have been identified. Dual luciferase reporter assays examined the binding between NNT-AS1, miR-889-3p, and HIF-1. After PDAC cells co-culture exosomes secreted by CAFs, we found that they alter glucose metabolism, proliferation, and metastasis. In PDAC cells, CAF-derived exosomal lncRNA NNT-AS1 acted as a molecular sponge for miR-889-3p. Furthermore, HIF-1 could be targeted by miR-889-3p and was controlled by NNT-AS1. This study explores the mechanism by which NNT-AS1 influences the interaction of CAFs on glycolytic remodeling, proliferation, and metastasis of tumor cells through regulating miR-889-3p/HIF-1α, which also helps discover new clinical treatment targets for PDAC.

## Introduction

Pancreatic ductal adenocarcinoma is one of the most aggressive gastrointestinal malignancies, with a 5-year survival rate of less than 10%^1^. Metabolic reprogramming, often known as the Warburg effect, is one of cancer’s ten hallmarks. It is important for cancer growth, including invasion and metastasis. Changes in the microenvironment can activate and maintain HIF, which, in turn, stimulates the transcription of related genes involved in metabolic reprogramming and PDAC generation, invasion, metastasis, and angiogenesis^2,3^. Meanwhile, cancer-associated fibroblasts (CAFs), a kind of activated fibroblast, are present in practically all solid tumors, especially in pancreatic, breast, and prostate cancers^4–6^. CAFs play a critical role in the formation and progression of a variety of cancers since they are one of the most important components of the tumor microenvironment (TME). Tumor cells and CAFs communicate in a variety of ways. It has been found that CAFs can aid cancer cells in acquiring energy, synthetic products, and a suitable environment to help cancer tissue adaptability, posing a challenge to anticancer therapy^7,8^.

Exosomes, a type of microvesicles, can modulate the TME by releasing signaling molecules into the extracellular environment (such as proteins, lipids, and nucleic acids)^9^. A broad group of transcripts with a length of more than 200 nucleotides is referred to as long noncoding RNAs (lncRNAs)^10^. They regulate cancer's proliferation, migration, invasion, and metabolic reprogramming via impacting DNA, chromatin, proteins, and microRNAs^11^.

Through the following processes, exosomal lncRNAs increase informational transmission between cancer cells and CAFs. To begin, it has been demonstrated that cancer-derived exosomes contain a large number of unique lncRNAs that are transported to the CAFs via microvesicles and regulate their function^12^. Meanwhile, CAFs can increase colorectal cancer stem cell properties and chemoresistance by conveying exosomal lncRNA H19 into colorectal cancer cells^13^. The CAFs-derived exosomal lncRNA SNHG3 serves as a molecular sponge for miR-330-5p in breast cancer cells, stimulating proliferation via metabolic reprogramming^14^. Finally, cancer-derived exosomal lncRNAs increase PDAC invasion and metastasis^15^. Nevertheless, the effects of lncRNAs from CAFs exosomes on pancreatic cancer cell formation, growth, and glycolytic reprogramming remain understudied,

NNT-AS1 is overexpressed in malignancies and promotes cancer growth by increasing cancer cell growth, migration, and invasion, as well as blocking apoptosis and cell cycle arrest^16–19^. We identified significant expression of NNT-AS1 in CAFs-derived exosomes and validated its influence on glucose metabolism in PDAC cells in our previous work. However, there is currently no evidence linking NNT-AS1 to pancreatic cancer or glucose metabolism. MiR-889-3p, which is weakly expressed in breast cancer, can prevent breast cancer cells from migrating^20^. Given the paucity of research on CAFs-derived exosomal lncRNA in PDAC, particularly to metabolic reprogramming, it's worth looking into the underlying molecular processes.

Based on the analysis, we hypothesized that the mechanism of NNT-AS1 influencing the interaction of CAFs and tumor cells via regulating miR-889-3p/HIF-1 should be thoroughly investigated from the standpoint of pancreatic cancer progression, and metabolic reprogramming.

## Materials and methods

### Tissue samples and cell culture

From 2020 to 2021, patients with pancreatic cancer and patients with pancreatic trauma were included in each. All patients had no prior radiation or chemotherapy and were confirmed by pathological examination. Fibroblasts were extracted from patients with pancreatic cancer and pancreatic trauma, respectively. The cells were processed with trypsin to separate them into individual cells and passaged according to 1:1. Non-fibroblasts such as pancreatic cancer cells and endothelial cells with late apposition were removed utilizing the differential apposition principle, and cell morphology tended to be uniform and absent of apparent senescence phenotypes by the 3rd-4th generation in passaged culture. Human pancreatic adenocarcinoma cells (Panc-1) and Human foreskin fibroblasts (HFF) were obtained from the Chinese Academy of Sciences (Shanghai, China). The cells were cultured in DMEM/F12 supplemented with 15% FBS. The CAFs were seeded and were extracted when 75% confluence was reached. Hubei Cancer Hospital Ethics Committees approved this study.

### Isolation and characterization of exosomes

CAFs were grown for 24 h in a low-glucose basal medium. Cells were grown in exosome-depleted serum. The cell supernatants were gathered and centrifuged at 400 × g for 5 min at 4 °C, following the cell supernatants were collected. And then, the supernatants were centrifuged at 3000 × g for 20 min at 4 °C to remove the dead cells debris. Next, the supernatants, which was generated by centrifuging at 100,000 × g for 1 h at 4 °C, were removed. Exosomes were generated by centrifuging again at 100,000 × g for 1 h at 4 °C. Enzyme-linked immunosorbent assay (Exosome Elisa Kit, Tingocell, China) was used to achieve quantitative exosome detection by detecting the exosome marker proteins CD9 and CD63. CAFs-derived exosomes were then added to the culture medium for continuous culture of pancreatic cancer cells Panc-1.

For our research, we utilized a transmission electron microscope to examine the morphology of the isolated exosomes (Hitachi HT7700, Japan). Size distribution of the separated exosomes was determined using qNano (Izon Science, New Zealand). The percentage of CAFs cells expressing for the molecular marker fibroblast activating protein (FAP) was determined using flow cytometry, and the fluorescence localization of α-SMA was determined using cellular immunofluorescence to further elucidate the cell phenotype. Anti-CD9 and anti-CD63 antibodies were utilized for the study of exosome markers in protein-based Western blotting.

### RNA extraction and quantitative real-time PCR (qRT-PCR) analysis

Researchers used TRIzol reagent (Invitrogen) to extract RNA from cells and exosomes, and then they reverse transcribed the cDNA to duplicate the RNA. NNT-AS1 siRNAs were created by Ribobio (Guangzhou, China). We bought miR-889-3p mimics and NC mimics from Ribobio. The primer sequences for NNT-AS1 were: 5′- CTG GAA TCC CTG CTA CTC AGG A -3′ (the forward primer) and 5′- GCC ATG TGA TAT GCC TGC TC -3′ (the reverse primer). The primer sequences for miR-889-3p were: 5′- ACACTCCAGCTGGGTTAATATCGGACAAC -3′ (the forward primer) and 5′-TGGTGTCGTGGAGTCG -3′ (the reverse primer). The primer sequences for β-actin were: 5′- CAG AGC AAG AGA GGC ATC C -3′ (the forward primer) and 5′- CTG GGG TGT TGA AGG TCT C -3′ (the reverse primer). The RNA primers were designed and synthesized by Ribobio. For quantitative real-time PCR, green SYBR qPCR was utilized (Takara Biotechnology, China). Normalizing miR-889-3p was done using U6 (the forward primer: 5′-CTCGCTTCGGCAGCACA-3′ and the reverse primer: 5′-AACGCTTCACGAATTTGCGT-3′), whereas normalizing NNT-AS1 was accomplished with β-actin. The relative change in RNA expression was determined using the 2^−△△C^ method.

### Luciferase reporter assay

After amplifying the section of NNT-AS1 sequences including the target-miR-889-3p binding sites, the products were inserted into the pmirGLO vectors (pmirGLO-NNT-AS1-WT). This plasmid was called NNT-AS1 WT. NNT-AS1 MUT was generated utilizing the target-miR-889-3p binding sites of NNT-AS1 as a template for PCR mutagenesis. Using the same approach, two HIF-1a constructs (one having the GAUAUUA binding sites, and the other carrying the CUAUAAU mutant binding sites) were created. Using Lipofectamine 2000, the Panc-1 cells were co-transfected mimics of miR-889-3p or NC mimics, along with either NNT-AS1 WT or NNT-AS1 MUT. We used pmirGLO-HIF-1a WT or pmirGLO-HIF-1a MUT together with miR-889-3p mimics or NC mimics in the same protocol. Luciferase activities were evaluated using a dual luciferase assay kit 48 h after cells were lysed (Promega).

### Vector constructs and transfections

The shRNA directed against NNT-AS1 was synthesized, reconstituted using pLVX-Puro vector, lentivirally packed, and transfected using lipofectamine 2000 (Invitrogen). Harvested logarithmic phase cells were plated onto 6-well plates. When cells reached about 80% confluence, they were transfected for 48 h with miR-889-3p mimics, miR-889-3p-inh, pc-HIF-1α (pc-DNA3.1-HIF-1α), or their equivalent controls.

### Western blot analysis

For western blots, we used the HIF-1 antibody from Santa Cruz Biotechnology and the LDHA, PKM2, SDH, FH, and β-actin antibodies from Cell Signaling. The immune complexes were identified using enhanced chemiluminescence after a further 1 h incubation with an anti-immunoglobin horseradish peroxidase-linked antibody (Cell Signaling Technology). All experiments were repeated three times. Due to the fact that our membranes were cropped, full-length membranes were not shown.

### Glucose uptake, lactate secretion, and mitochondrial immunofluorescence staining

Cells were grown for 16 h in glucose-free DMEM and then incubated for an additional 24 h in high-glucose DMEM. The glucose uptake and lactate secretion were performed using the Glucose Assay Kit (JianCheng, NanJing, China) and the Lactate Assay Kit (JianCheng, NanJing, China), respectively. Three times were repeated per experiment. Inverted fluorescence microscopy was used to detect mitochondrial activity in Panc-1 cells after 24 h of incubation and immediately after 30 min of incubation with DMEM containing mitochondrial fluorescent probes.

### CCK-8 assay

Cells were seeded at a density of 3 × 10^3^/well in 96-well plates. The CCK-8 test was used by measuring the absorbance OD at 450 nm with a microplate spectrophotometer (Molecular device, USA).

### Wound healing assay

Six-well plates were prepared, with 1 × 10^6^ cells planted, and cultured. Cell monolayers were scratched using a 200 ml plastic pipette tip, which produced wounds, and then cultured in new media for 24 h. Measuring the average number of moving cells per field was done using photographs.

### Cell migration assay

Invasion assays were conducted using 8.0 μm pores in a Trans-well insert. A cell suspension of 1 × 10^5^ cells was introduced to the top chamber of a trans-well, and the mixture was incubated overnight. 1/6 diluted matrigel filter media was used. The bottom chamber was mixed with DMEM containing 10% fetal bovine serum and left to incubate for 24 h. Paraformaldehyde was used to fix the migratory cells, after which they were stained with crystal violet at a concentration of 0.1 percent. Each of the five target fields was scrutinized using a 20× objective.

### Bioinformatics analysis

DIANA (http://diana.imis.athena-innovation.gr/DianaTools/index.php) database for NNT-AS1 and miR-889-3p binding prediction. STARBASE (http://starbase.sysu.edu.cn/index.php) database for predicting HIF-1 and miR-889-3p binding.

### Statistical analysis

The mean and standard deviation (SD) of at least three separate experiments are presented. We used the Student’s t-test to analyze the data. A *P* < 0.05 value was deemed significant. SPSS 20.0 was used to conduct all statistical analyses.

### Ethics approval and consent to participate

This research was approved by the Clinical Research Ethics Committees of Hubei Cancer Hospital (LLHBCH2021YN-028). All samples were obtained with informed consent. Participants provided written informed consent prior to taking part in the study. The study was performed in according with the ethical standards as laid down in the 1964 Declaration of Helsiniki and its relevant enical standards.

## Results

### Effect of CAFs-derived exosomes on the metabolism of PDAC cells and validation of their NNT-AS1 expression levels

**Figure 1:**
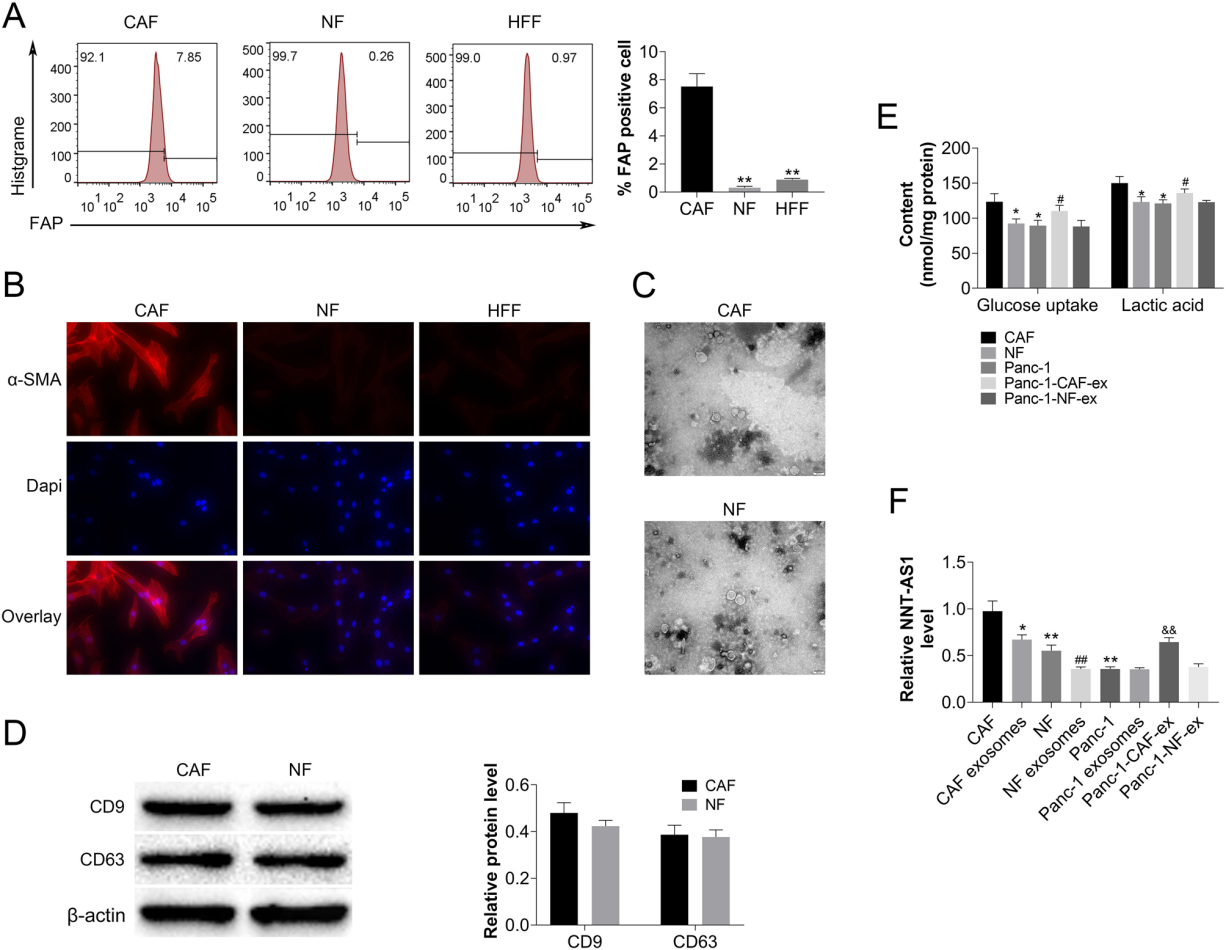
Characterization of CAFs-derived exosomes, their influence on glucose metabolism, and their enhanced expression of NNT-AS1. (**A**) Flow cytometry was used to measure the proportion of CAFs molecular marker FAP-positive cells in a cohort (compared with CAF group, **P < 0.01). (**B**) Detection of -SMA fluorescence localization and clarification of its cellular phenotype using cellular immunofluorescence. (**C**) Cup-shaped exosomes as seen using transmission electron microscopy. (**D**) The expressions of CD9 and CD63 in CAFs-derived exosomes and controls were examined using Western blot. (**E**) Panc-1 glucose metabolism changes following mixing with CAFs or NF isolated exosomes in culture, as measured by glucose absorption and lactate secretion(Compared with Panc-1 group, * *P* < 0.05). (**F**) After mixing Panc-1 with CAFs or NF isolated exosomes in culture, a qRT-PCR analysis in corresponding cells or exosomes was used to examine the change in NNT-AS1 expression. (E) Changes in glucose uptake and lactate secretion of Panc-1 after mixed culture with exosomes isolated from CAF or NF (compared with CAF group, *P < 0.05; Compared with Panc-1 group, #*P* < 0.05). (F) After mixed culture of Panc-1 and exosomes isolated from CAFs or NF, qRT-PCR analysis was performed in the corresponding cells or exosomes to examine the change of NNT-AS1 expression (compared with CAF group, *P < 0.05 and **P < 0.01; Compared with CAF exosomes group, ##*P* < 0.01; Compared with Panc-1 group, &&*P* < 0.01).

Primary cancer-associated fibroblasts (CAFs) from PDAC tissues and normal fibroblasts (NF) from pancreatic trauma patient tissues were isolated and purified, and CAFs were identified and tested for purity by phenotype and marker protein. The proportion of the CAFs molecular marker fibroblast activating protein(FAP)-positive cell population was detected by flow cytometry (Fig. 1A), and a myofibroblast marker (α-SMA) was detected by a cellular immunofluorescence technique to clarify the cell phenotype (Fig. 1B). Human foreskin fibroblasts (HFF) were also cultured in vitro as a control. Transmission electron microscopy showed that these exosomes have a typical cup-like bilayer (Fig. 1C). The analysis of the particulate size indicated that the vesicle diameter was within the exosome diameter. Markedly unregulated expression of exosomal markers CD9 and CD63 proteins were identified by protein blotting (Fig. 1D). CAFs-derived exosomes could increase glucose uptake and lactate secretion in Panc-1 cells, whereas NF-derived exosomes did not (Fig. 1E). Real-time quantitative qRT-PCR of RNA extracted from cells or exosomes revealed that, compared to NF-derived exosomes, CAFs-derived exosomes increased the lncRNA NNT-AS1 content of Panc-1 cells (Fig. 1F).

### The effects of the CAFs-derived exosomes NNT-AS1 on PDAC cell progression, and glycolytic capability

**Figure 2:**
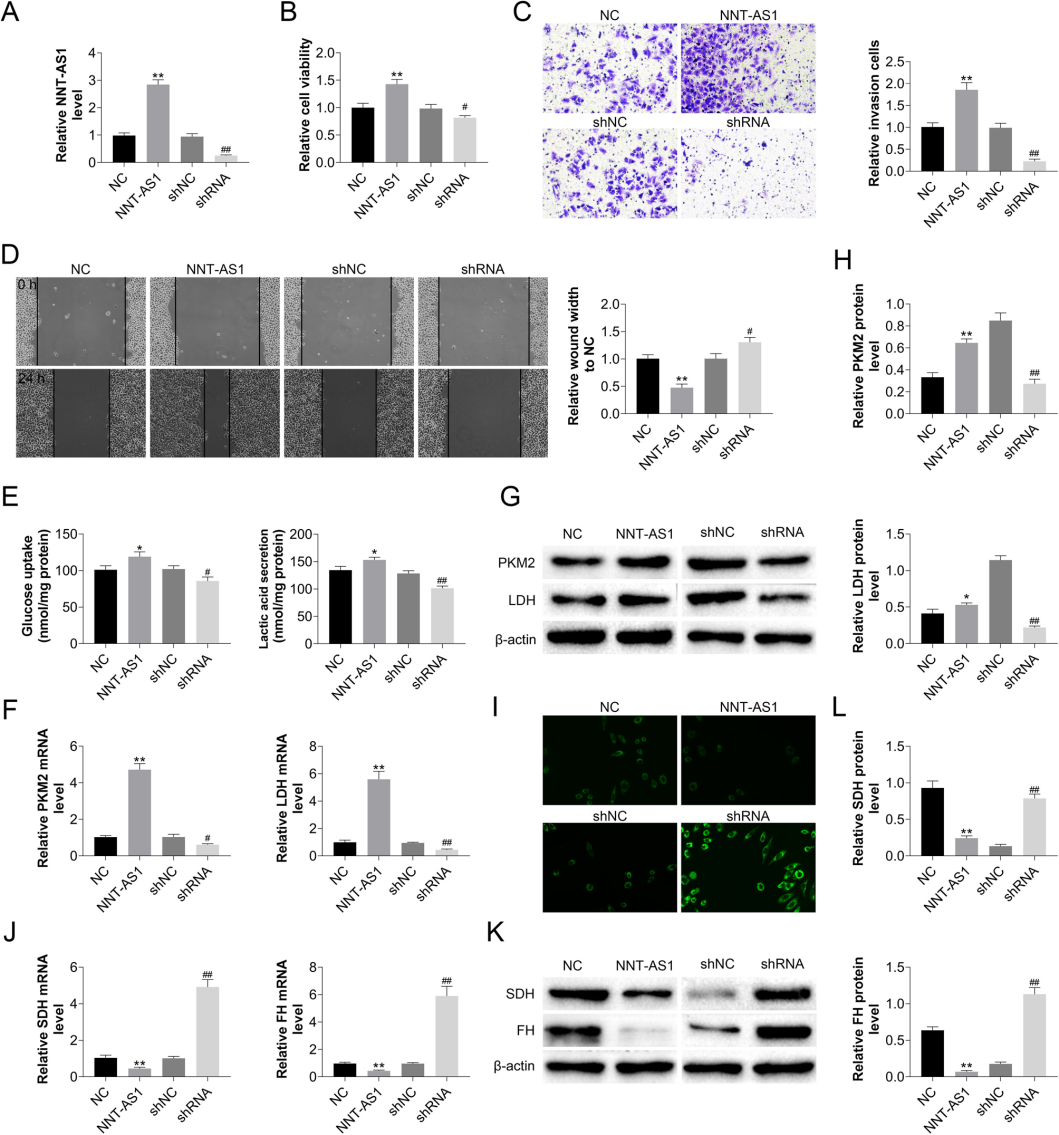
NNT-AS1 in CAFs-derived exosomes increases PDAC cell motility, invasion, proliferation, and glucose metabolism reprogramming. (**A**) Exosomes were isolated from CAFs cell lines that were trans-silenced/overexpressing NNT-AS1, and NNT-AS1 expression was confirmed by PCR. (**B**) CCK8 analyzed how the amount of NNT-AS1 in CAFs-derived exosomes affected the viability of PDAC cells. (**C**) The invasion of PDAC cells varied with the amount of NNT-AS1 in CAFs-derived exosomes, according to a transwell assay. (**D**) Experiments on wound healing confirmed that the CAFs-derived exosomes improved the motility of PDAC cells. (**E**) Glucose uptake and lactate secretion of these cells were quantified and normalized for cellular protein content. qRT-PCR and Western blot were used to assess PKM2 and LDH (two key enzymes in anaerobic glycolysis) (**F–H)**; inverted fluorescence microscopy was utilized to investigate changes in mitochondrial activity, and SDH and FH (two key enzymes in aerobic glycolysis) were detected using qRT-PCR and Western blot (**L–K**). (Compared with NC, NNT-AS1 group (NNT-AS1 overexpression group) had *P < 0.05 or **P < 0.01 statistical difference; Compared with the shNC group, the shRNA group (NNT-AS1 silenced group) had a statistical difference of #*P* < 0.05 or ##*P* < 0.01).

Exosomes were extracted from CAFs that had been stably transfected with overexpressed or silenced NNT-AS1, and PCR assays revealed corresponding changes in NNT-AS1 (Fig. 2A). After CAFs cells were silenced or overexpressed with NNT-AS1, exosomes were extracted, mixed with Panc-1, and incubated. Changes in PDAC cell proliferation were observed using the CCK-8 assay (Fig. 2B). The influence of PDAC cell invasion ability was observed using the transwell assay (Fig. 2C), and the impact of PDAC cell migration ability was measured using the scratch assay (Fig. 2D). NNT-AS1 in CAFs-derived exosomes significantly increased the proliferation, invasion, and motility of PDAC cells. Cells were cultured as above, in addition, the effect of NNT-AS1 on glycolytic ability was confirmed by glucose absorption and lactose secretion (Fig. 2E), while the expression levels of two key enzymes (M2-type pyruvate kinase, PKM2) and lactate dehydrogenase, LDH)) in the anaerobic glycolysis process were evaluated using qRT-PCR and Western blot (Fig. 2F–H); after incubation with DMEM containing mitochondrial fluorescent probes (Fig. 2I), inverted fluorescence microscopy was used to observe mitochondrial activity, while the expression levels of two key enzymes (succinate dehydrogenase (SDH) and fumarate hydratase (FH)) in the tricarboxylic acid cycle were detected using qRT-PCR and Western blot (Fig. 2L–K). In PDAC cells, NNT-AS1 in CAFs-derived exosomes boosted anaerobic glycolytic ability while decreasing aerobic glycolytic capacity; inhibiting NNT-AS1 expression restored the glycolytic alterations.

### NNT-AS1 in CAFs-derived exosomes negatively regulated miR-889-3p in PDAC cells, and the effect of miR-889-3p on PDAC cell progression, and glucose metabolism

**Figure 3:**
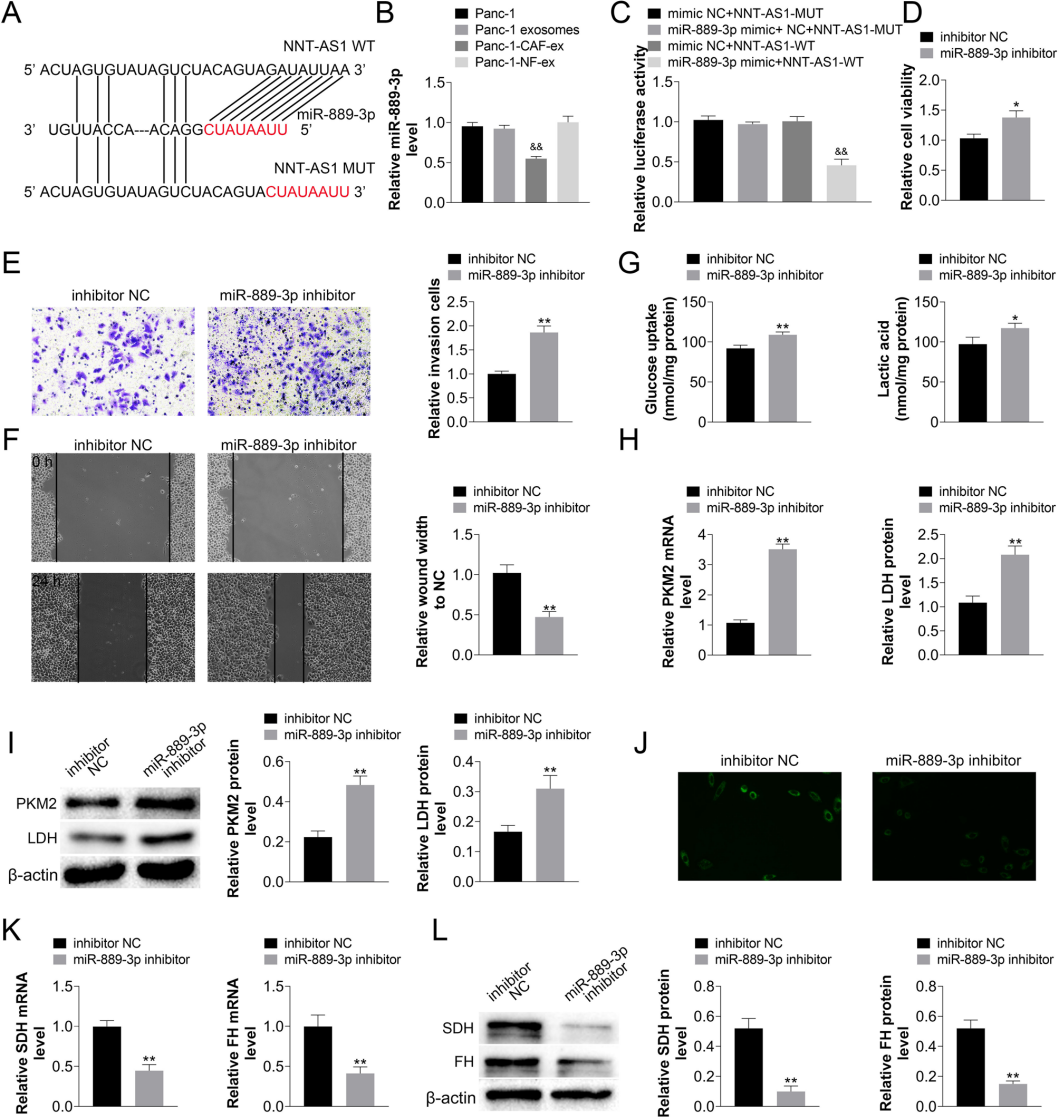
The suppressed effect of miR-889-3p on PDAC cell proliferation, metastasis, and glucose metabolism reprogramming was due to NNT-AS1 in CAFs-derived exosomes inhibition miR-889-3p in PDAC cells. (**A**) The DIANA database was used to predict NNT-AS1 and miR-889-3p binding sites. (**B**) Panc1 was co-cultured with exosomes obtained from CAFs cell lines, and PCR was used to validate the expression alterations of miR-889-3p in PDAC (Compared with Panc-1 group, **P < 0.01). (**A**, **C**) For the Luciferase reporter gene test, recombinant wild type (NNT-AS1-WT) and mutant reporter constructs (NNT-AS1-MUT) were created. In Panc1 cells, co-transfected with NNT-AS1-WT or NNT-AS1-MUT and miR-889-3p-mimics or mimic NC, the luciferase activity was evaluated (Compared with mimic NC + NNT-AS1-W group, **P < 0.01). Panc1 cells were successfully transfected with the miR-889-3p inhibitor. After knocking down miR-889-3p, the CCK-8 assay showed alteration proliferation ability (**D**); the transwell assay showed changes in cell invasion ability after miR-889-3p downregulation (**E**); cell motility was observed in a scratch assay (**F**); and qRT-PCR and Western blot for PKM2, LDH, SDH, and FH, as well as quantification of glucose uptake, lactose secretion, and mitochondrial activity, were used to examine reprogramed glucose metabolism in Panc-1 cells (**G–L**). (Compared with inhibitor NC group, miR-889-3p inhibitor group had statistical difference of *P < 0.05 or **P < 0.01).

NNT-AS1 and miR-889-3p binding sites were predicted using the DIANA database (Fig. 3A). Meanwhile, the decrease in miR-889-3p expression in PDAC cells cultured by CAFs-derived exosomes was also detected using qRT-PCR (Fig. 3B). Dual-luciferase vectors were constructed utilizing partial sequences containing binding sites and modified matching sequences, and the binding areas of NNT-AS1 and miR-889-3p were validated using a dual-luciferase reporter gene experiment (Fig. 3C). The miR-889-3p inhibitor was transfected into PDAC cells, and the CCK-8 assay revealed that knocking down miR-889-3p increased PDAC cell proliferation (Fig. 3D), while the transwell assay revealed that miR-889-3p down-regulation increased PDAC cell invasion (Fig. 3E), and the scratch assay revealed that PDAC cell migration increased after miR-889-3p silencing (Fig. 3F). PKM2, LDH, SDH, and FH were assessed by qRT-PCR and Western blot, as well as glucose absorption, lactose secretion, and mitochondrial activity were quantified (Fig. 3G–L). In PDAC cells, knocking down miR-889-3p boosted anaerobic glycolysis while downregulating aerobic metabolism. Changes in the expression of critical genes involved in glucose metabolism were also detected. These findings imply that NNT-AS1 in CAFs-derived exosomes inhibited miR-889-3p in PDAC cells and that miR-889-3p down-regulation increased PDAC cell progression, and anaerobic glycolysis.

### HIF-1α, a miR-889-3p target, correlated favorably with NNT-AS1 in CAFs-derived exosomes and facilitated progression, and glucose metabolism reprogramming in PDAC cells

**Figure 4:**
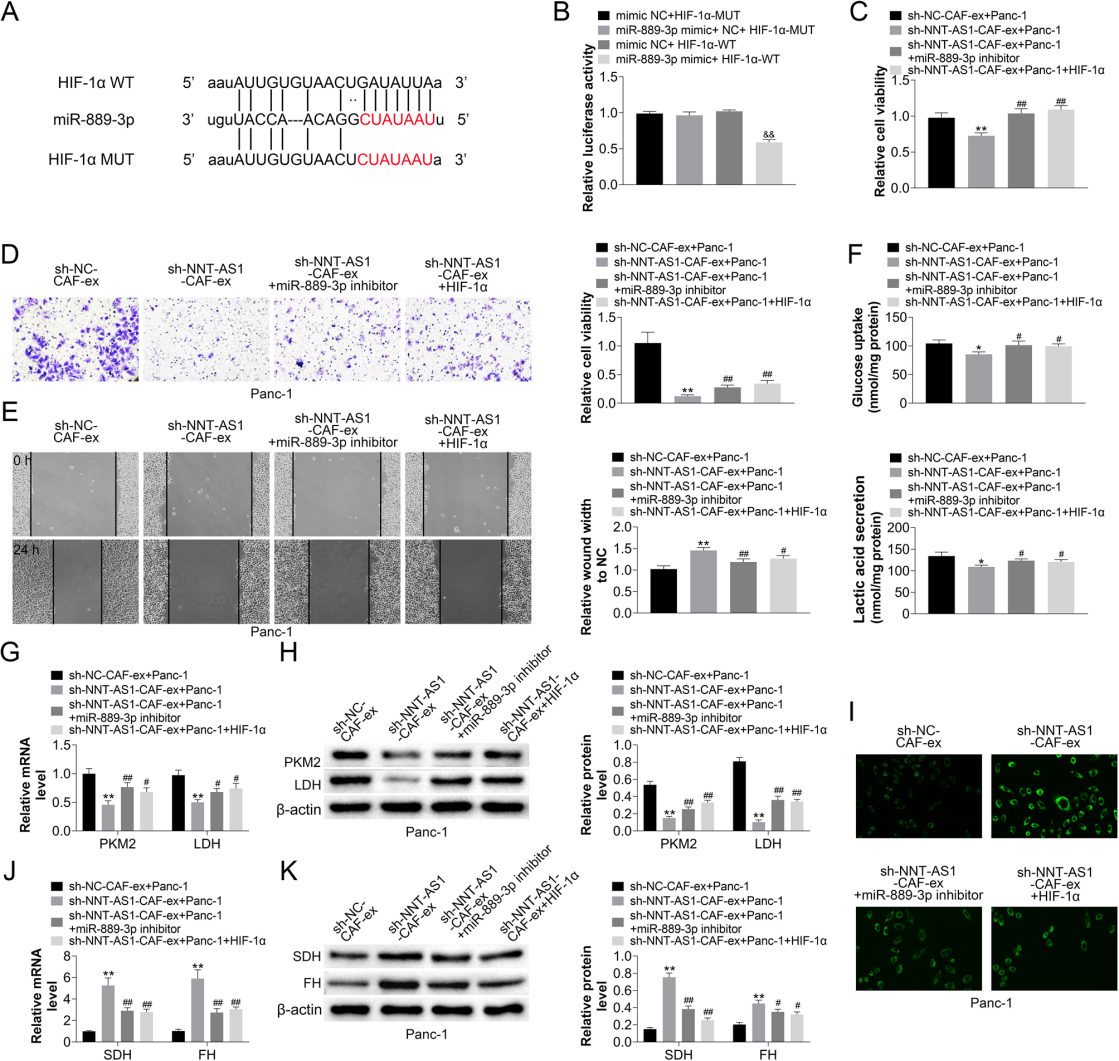
HIF-1α, a miR-889-3p target, is favorably correlated with NNT-AS1 in CAFs-derived exosomes. (**A**) The prediction for miR-159-3p binding sites on HIF-1α was discovered by bioinformatics analysis. (**B**) The binding site and its altered matching sequences were initially generated, and the plasmids were recombinant and co-transfected with miR-889-3p mimic or mimic NC. After 48 h of incubation, the direct binding of HIF-1α and miR-889-3p was confirmed by determining the ratio of fluorescent firefly luciferase activity to sea kidney luciferase (compared with mimic NC+HIF-1α--WT group, **P < 0.01). Exosomes were extracted from NNT-AS1-silenced CAFs cells and treated for 24 h with Panc-1 cells transfected with miR-889-3p inhibitor or HIF-1 over-expression plasmids. (**C**) CCK-8 test was utilized to evaluate the changed effect of miR-889-3p inhibitor or overexpression of HIF-1α on the inhibitory impact of low expression of NNT-AS1 in CAFs-derived exosomes on the proliferation of PDAC cells. (**D, E**) Transwell and scratch studies were carried out to see if a miR-889-3p inhibitor or HIF-1 overexpression might counteract the effect of reducing NNT-AS1 in CAFs-derived exosomes on PDAC cell invasion ability. (**F–I**) To investigate changed glucose metabolism in Panc-1 cells, qRT-PCR and Western blot for PKM2, LDH, SDH, and FH, as well as measurements of glucose uptake, lactose secretion, and mitochondrial activity, were utilized. (Compared with sh-NC-CAF-ex+Panc-1 group, *P < 0.05 and **P < 0.01; Compared with SH-NNT-AS1-CAV-EX+Panc-1 group, #*P* < 0.05 and ##*P* < 0.01).

Predicting the binding region of HIF-1α and miR-889-3p using the Starbase database (Fig. 4A). Dual-luciferase reporter gene assays were utilized to confirm the areas with direct binding of HIF-1 and miR-889-3p after reconstitution of plasmids utilizing mutagenesis and gene recombination procedures (Fig. 4B). Using the CCK-8 experiment, we discovered that miR-889-3p inhibitors or HIF-1α overexpression may reverse the effect of reduced NNT- AS1 expression in CAFs-derived exosomes on PDAC cell proliferation inhibition (Fig. 4C). Further, the detection of miR-889-3p inhibitors or HIF-1α overexpression reversed the inhibitory impact of low NNT-AS1 expression in CAFs-derived exosomes on PDAC cell invasion ability utilizing transwell and scratch assays (Fig. 4D, E). In addition, the anaerobic glycolytic and aerobic metabolic capabilities, as well as the levels of expression of important genes, were investigated. The inhibitor of miR-889-3p or overexpression of HIF-1α reversed the inhibitory impact of CAFs-derived exosomes silencing NNT-AS1 upon Panc-1 anaerobic glycolysis and the facilitative impact on aerobic metabolism (Fig. 4F–I).

## Discussion

Tumor microenvironment-induced resistance to therapy develops in addition to intrinsic resistance resulting from genetic and epigenetic alterations in tumor cells. Tumor microenvironment (TME): a vascularized stroma containing a complex network of normal (non-neoplastic) and neoplastic cells. The primary constituents of the TME are cancer-associated fibroblasts (CAFs), one of the many stromal cell types^8^. Pancreatic cancer is a malignant tumor with a poor blood supply that occupies more than 90% of the tumor volume^21^. The high stromal component increases pancreatic cancer growth and progression and also impairs the cytotoxic impact of chemotherapeutic medicines on pancreatic tumor cells^22^. Nab-paclitaxel is a first-line drug for pancreatic cancer treatment primarily because it can diminish cancer-associated fibrosis and cancer’s stromal component^23^. Additionally, tumor cells can acquire energy and generate products when provided with an appropriate environment, but this also presents a difficulty to cancer treatment^7,8^. Like a group of nanovesicles, exosomes have a certain class of molecules and are especially abundant in protein, nucleic acid, and lipid contents. A huge series of researches have all but concluded that exosomes have a key role in cancer, and may aid future diagnoses and treatments. It is uncertain, however, which specific molecular pathways underlie these effects, as well as whether they are useful in therapeutic settings. Because of its abundance in the tumor stroma, CAFs can enable the development and metastasis of cancer. Multiple investigations have found that CAFs are metabolically cooperative with tumor cells. Through their secretion of growth factors, chemokines, cytokines, and exosomes, CAFs have a significant impact on the progression and spread of tumors. Tumor cells and TME cells can communicate through the production of exosomes. The reciprocal contact between surrounding non-tumoral cells and malignant cells is facilitated by exosome-mediated cell signaling^10,11^. Immersive research on CAFs in pancreatic cancer has emerged as a new therapeutic method for metastasis^24^.

While no cell surface indicators have yet been found that can identify CAF, several publications are stating that it can be detected by biomarkers, such as a-SMA, FAP, and cell morphology^25^. Our study agrees with previously conducted research, where CAF showed greater levels of FAP and a-SMA to NF.

As of this writing, an abundance of research has documented that lncRNAs regulate proliferation and apoptosis in various tumor cells, including pancreatic cancer, by working in concert with HIF-1α to modulate cell survival and apoptosis. In the context of pancreatic cancer, the study of CAFs-derived exosomal lncRNA function has thus far been scarce. According to previous studies, CAFs-derived exosomes accelerate breast cancer cell growth by positively modulating metabolism^14^. However, the results from the study about exosomes derived from pancreatic cancer, and their influence on cancer cell glucose metabolism, have not been thoroughly researched.

NNT-AS1 is highly expressed in cancer and helps tumor cells to proliferate, migrate, and invade. This boosts the possibility of cancer formation, including breast, lung, and gastric cancers. However, the connection between NNT-AS1 and glycolytic remodeling in pancreatic cancer was previously unclear. We demonstrate in this study that NNT-AS1 expression is present in pancreatic cancer patient-derived CAFs. Suppression of CAFs-derived exosomal NNT-AS1 reduces pancreatic cancer cell progression, and glucose metabolism reprogramming. NNT-AS1 could act as a molecular sponge for miR-889-3p, inhibiting its expression. miR-889-3p was able to partially reverse the effect of exosomal NNT-AS1 treatment, implying that exosomal NNT-AS1 may be involved in the regulation of the expression of additional potential miRNAs. the 3′UTR region of HIF-1α is targeted by miR-889-3p, which in turn suppresses HIF-1α production. Increased miR-889-3p expression partially reversed the effect of CAFs-derived exosomal NNT-AS1, implying that exosomal NNT-AS1 may be involved in the regulation of the production of other possible miRNAs. These findings shed light on the biological role and molecular control of NNT-AS1 in CAFs for the first time.

However, this study leaves several concerns unanswered, and the regulatory mechanism by which the lncRNA NNT-AS1 expression level is regulated in CAFs and how it is paracrine needs to be developed further.

This research proves the expression pattern of exosomal lncRNA NNT-AS1 in CAFs, ascertains how it acts on HIF-1α to influence glucose metabolism, proliferation, and metastasis of PDAC cells, and reveals how it regulates metabolic reprogramming, cell proliferation, and metastasis in pancreatic cancer cells. Through the prospecting of miRNAs and lncRNAs, as well as their roles in the HIF-centered metabolic co-regulation process, it may be possible to discover new information about lncRNAs and how they regulate specific tumor malignancies. Identifying the role of CAFs might highlight the significance of stromal signature prognostic and therapeutic prediction in cancer. This new information may also help with earlier diagnosis and treatment of PDAC (Supplementary Figures).

While the majority of research to date has concentrated mostly on tumor cells, communication within the tumor microenvironment (TME) through exosome-mediated crosstalk and direct cellular interaction is also linked to the malignant characteristics of malignancies. Consequently, a deeper comprehension of the process driving interaction between cancer cells and the TME throughout therapy is required.In summary, exosome-mediated communication between tumor cells and tumor-associated stromal cells promotes tumor growth and resistance to treatment, indicating the need for novel cancer treatment approaches^8^.

## Conclusion

In conclusion, overexpression of lncRNA NNT-AS1 in CAFs-derived exosomes acts as a miR-889-3p sponge in PDAC cell, regulating HIF-1 expression, enhancing PDAC cell proliferation and metastasis, and reprogramming glucose metabolism.

### Supplementary Information

Supplementary Figures.

## Data Availability

The datasets used and/or analysed during the current study available from the corresponding author on reasonable request.

## Abbreviations

NNT-AS1: Nicotinamide nucleotide transhydrogenase-antisense RNA1
PDAC: Pancreatic ductal adenocarcinoma
CAFs: Cancer-associated fibroblasts
NF: Normal fibroblasts
FAP: Fibroblast activating protein
TME: Tumor microenvironment
LncRNA: Long noncoding RNA
CCK-8: Cell counting kit-8
HIF-1: Hypoxia inducible factor 1
LDHA: Lactate dehydrogenase A
PKM2: M2-type pyruvate kinase
SDH: Succinate dehydrogenase
FH: Fumarate hydratase
OD: Optical density
SD: Standard deviation
